# The Significance of Serum Phosphate Level on Healing Index and Its Relative Effects in Skeletally Immature and Mature Patients with Hypophosphatemic Rickets

**DOI:** 10.1155/2014/569530

**Published:** 2014-04-24

**Authors:** Sang-Heon Song, Hanna Lee, Ji-Min Jeong, Woo-In Cho, Sung Eun Kim, Hae-Ryong Song

**Affiliations:** ^1^Department of Orthopaedic Surgery, Myongji Hospital, 55 Hwasu-ro 14 Beon-gil, Deokyang-gu, Goyang 412-826, Republic of Korea; ^2^Department of Orthopaedic Surgery and Rare Diseases Institute, Korea University Medical Center, Guro Hospital, 80 Guro-dong, Guro-gu, Seoul 152-703, Republic of Korea; ^3^Department of Orthopaedic Surgery, Soonchunhyang University Bucheon Hospital, 170 Jomaru-ro, Wonmi-gu, Bucheon, Gyeonggi-do 420-767, Republic of Korea

## Abstract

The aim of this study was to find out the ideal cut-off level of phosphate for safe healing when deformity correction and concomitant lengthening are indicated in the two different skeletal maturity groups of patients with rickets. Thirty-nine hypophosphatemic rickets patients were selected for the study and were divided into two groups: 27 skeletally immature (group IM) and 12 skeletally mature (group M). The outcomes were evaluated with respect to the healing index (HI), laboratory findings, and complications with the mean follow-up of 5.1 years (range, 3.1–7.9). The healing index (HI) of group IM was 1.44 month/cm and HI of group M was 1.68 month/cm. The negative correlation between the level of serum phosphate and HI in group M (coefficient = −0.94) was evaluated to be less than the correlation in group IM (coefficient = −0.50), indicating that the HI is more likely to be affected by serum phosphate in group M than in group IM. Preoperative serum phosphate levels of 2.3 mg/dL and 2.6 mg/dL were analyzed to be the cut-off values of group IM and group M, respectively, in which the cut-off points divided the series into two groups having the most significantly different HI.

## 1. Introduction

Rickets is a group of diseases characterized by deficient or impaired mechanism of vitamin D due to a variety of etiologies. One of the most crucial clinical features is hypophosphatemia, which leads to poor bone mineralization and consolidation, resulting in limb deformities and short stature starting at young age. Low level of phosphate impairs phosphate-dependent apoptosis of hypertrophic chondrocytes and is considered the molecular basis for rickets. Phosphate plays an important role in the skeleton that extends beyond mineralized matrix formation and growth plate maturation and is critical for endochondral bone repair [1].

Previous authors reported the surgical outcomes of deformity correction and variable affecting factors in hypophosphatemic rickets [2–4]. And one of them [2] reported the significance of serum phosphate level in deformity correction of the knee and leg lengthening by Ilizarov method in hypophosphatemic rickets. They concluded that a serum phosphate level of 2.5 mg/dL should be considered as the cut-off level in deciding whether a patient may be considered to undergo the procedure for leg lengthening in their heterogenic age group. However, some authors reported that age factors can also affect the healing index and consolidation time [4, 5].

So we hypothesized that the optimal serum phosphate level for deformity correction and concomitant leg lengthening would be different for skeletally immature and mature patients and aimed to find the ideal cut-off level of phosphate for each group of skeletal maturity.

## 2. Materials and Methods

With the approval of the review board of our institute, we retrospectively reviewed the history of a total of 61 patients who were diagnosed with hypophosphatemic rickets between the years 2002 and 2008. Diagnosis was made by the presence of radiologic findings, such as long bone cupping, splaying, and fraying of the metaphyseal areas and lab values such as elevated alkaline phosphatase level, low 25-OH vitamin D, and low percent tubular reabsorption of phosphate (TRP) [6, 7]. Among the 61 patients who underwent corrective osteotomy and lengthening on both femur and tibial segment or either, only tibial segments were enrolled to minimize affecting variables and bias for this study. Patients who were not followed up for more than 3 years after the initial operation were excluded. Patients who underwent corrective osteotomy alone without limb lengthening were also excluded from the study owing to the fact that the focus of our study was mainly on the relationship of phosphate and healing index in rickets patients with limb lengthening in addition to deformity correction. Also the patients who needed triple osteotomy were excluded.

Upon inclusion and exclusion of patients with respect to the criteria mentioned above, 39 patients were selected for this study. The patients were then divided into two main groups according to the physeal closure patterns of knee radiographs—27 skeletally immature (group IM) and 12 skeletally mature (group M) patients. The mean ages of the patients in group IM and group M were 9.6 years (range: 3.8–15.8) and 23 years (range: 18.3–31.2) at the time of surgical treatment, respectively. The mean durations of follow-up of group IM and group M after initial treatments were 5.4 years (range: 3.1–7.6) and 4.8 years (range: 3.6–7.9). Only one side of the tibia of a patient was randomly selected and considered as one sample segment to minimize any bias caused by correlation of bilateral tibia of a patient [8]. Therefore, a total of 39 tibiae of 39 rickets patients were enrolled for this study.

Corrective osteotomies were performed at the single, double CORA levels. The Ilizarov ring fixator was used for gradual or acute correction in all tibial deformities. The decision of performing concomitant leg lengthening with deformity correction was made after a thorough discussion with the patients and their families. Distraction was started 7 days postoperatively at a rate of 0.25 mm four times a day until the normalization of the mechanical axis and the desired length was achieved. The distraction rate was controlled according to the degree of callus formation. The external fixator was removed upon visualization of callus formation in 3 cortices at the distraction site [9, 10].

The skeletally immature patients had been under medical treatment of hypophosphatemic rickets, which included anhydrous phosphate (Joulie's) oral solution (average intake of 15 mL per dose, five times daily) along with vitamin D or its metabolites. Joulie's solution was omitted in the morning of operation and was readministered as soon as bowl function returned after the operation. 1, 25-Dihydroxyvitamin D (0.025–0.050 *μ*g/kg per day) was stopped about a week before surgery and was readministered when mobilization of the leg became possible, usually 5 to 7 days after the operation [2, 11].

The tibiofemoral angle (TFA) and mechanical axis deviation (MAD) were measured using the radiographic images that were taken before the operation, at the time of removal of the external fixator and at the final follow-up. Additionally, the knee joint alignment was assessed using Fraser et al.'s criteria [12] and classified into three: excellent (TFA valgus 5–9° in female, valgus 4–7° in male), good (within 4° from the normal range), or poor (out of 4° from the normal range). Length gain was calculated from the uppermost portion of the tibial eminence to the midpoint of the lowermost portion of the tibial plafond in the orthoroentgenograms. Healing index (HI) was computed by dividing the number of months for the operated segment to union by the final regenerate length gain.

The laboratory values of calcium, phosphate, and alkaline phosphatase (ALP) were assessed for biochemical evaluation.

Complications were evaluated and classified into major and minor according to Paley's classification. The major complication did interfere with the original goals of treatment and the minor did not [13]. Also they were classified into bony and other complications. Any osteotomy losing more than 10° from the immediate postoperative position in either the AP or the valgus-varus plane was considered a recurrence of the deformity [14].

All statistical analyses were performed using SPSS (SPSS for Windows Release 12.0; SPSS Inc., Chicago, IL, USA). The Mann-Whitney *U* test was used for data from two groups. The correlations between HI and other factors such as age and serum phosphate level were analyzed using linear regression model. The receiver operating characteristic (ROC) curve analysis was performed to determine the cut-off point in preoperative serum phosphate level by which the lengthened bone segments could be divided into two groups having the most significantly different HI.

## 3. Results

In group IM, 24 patients had bilateral genu varum deformity and 3 patients had bilateral genu valgum deformity. In patients with genu varum deformity, the mean TFA averaged 20° (range: 2° to 36°) prior to the operation, 5° (range: 2° to 19°) at the time of removal of external fixator, and 6° (range: −8° to 22°) at the time of final follow-up. Among these patients, MAD averaged 3.5 cm (range: 4.4 to 5.8), 0.9 cm (range: −0.6 to 2.5), and 1.7 cm (range: −1.5 to 4.2) of the center of the knee joint preoperatively, at the time of external fixator removal, and at the time of final follow-up. Among the 3 patients who had bilateral genu valgum deformity in the IM group, TFA averaged −10° (range: −12° to −9°), −4.° (range: −11° to 1°), and 0° (range: −4° to 2°), and MAD averaged −16.0 cm (range: −20.0 to −14.0), −4.8 cm (range: −17.6 to 7.8), and 1.9 cm (range: −6.4 to 6) preoperatively, at the time of external fixator removal, and at the time of final follow-up.

In group M, 10 patients showed genu varum deformity and 2 patients showed genu valgum deformity. Among patients with genu varum deformity, the TFA averaged 16° (range: 1° to 26°), 6° (range: −5° to 12°), and 6° (range: −5 to 17), and MAD averaged 4.0 cm (range: 0.4 to 8.3), 1.7 cm (range: −0.4 to 4.8), and 1.3 cm (range: −1.8 to 2.8) preoperatively, at the time of external fixator removal, and at the time of final follow-up. In two patients with bilateral genu valgum deformity, the TFA averaged −9° (−12° and −5°), 3° (−3° and 8°), and 1° (−5 ° and 6 °), and MAD averaged −2.1 cm (−3.0 and −1.2), 0.2 cm (−0.6 and 1.0), and −0.2 cm (−0.4 and 0.0) preoperatively, at the time of external fixator removal, and at the time of final follow-up (Table 1).

The mean healing indices of group IM and group M were 1.44 months/cm (range: 0.71 to 2.52) and 1.68 months/cm (range: 0.85 to 2.57), respectively, yielding statistically significant difference (*P* = 0.04). The mean amounts of the lengthening were 4.43 cm (range: 1.70 to 10.46) and 4.96 cm (range: 2.14 to 8.33), respectively, without statistically significant difference (*P* = 0.56) (Table 2).

Preoperative laboratory values such as phosphate, ALP, and calcium levels were evaluated and statistically analyzed. The mean levels of phosphate showed 2.58 mg/dL (range: 1.90–3.54) and 2.21 mg/dL (range: 1.73–2.84) in group IM and group M (*P* = 0.01), and the mean ALP levels were 574.96 U/IL (range: 352–1022) and 149.50 U/IL (range: 85–265), respectively, (*P* = 0.01). The mean levels of calcium were 9.32 mg/dL (range: 8.73–9.82) and 10.03 mg/dL (range: 9.14–13.14) and showed no statistical difference (*P* = 0.25) (Table 3).

**Figure 1:**
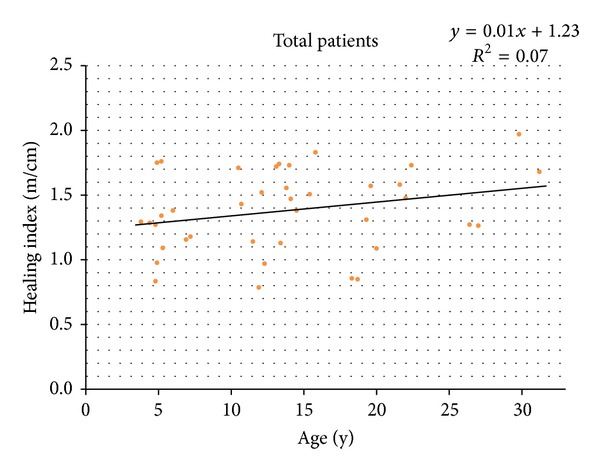
The correlation between age and healing index shows a positive linear pattern, but there was no statistical significance (*P* = 0.11).

The correlation between healing index and age of the patients was evaluated. Even though the relationship between HI and age showed a linear pattern, there was no statistically significant correlation between the two parameters in linear regression model (*P* = 0.11) (Figure 1). Also, the correlation between the sex of the patient and his/her healing index did not show statistical significance (*P* = 0.86).

**Figure 2:**
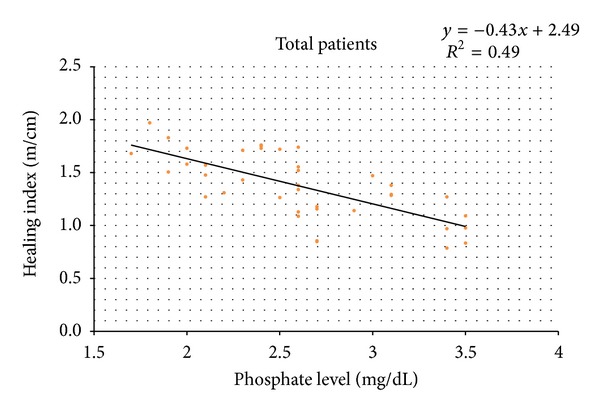
The correlation between serum phosphate level and healing index shows a significant negative linear regression pattern (*P* = 0.01).

**Figure 3:**
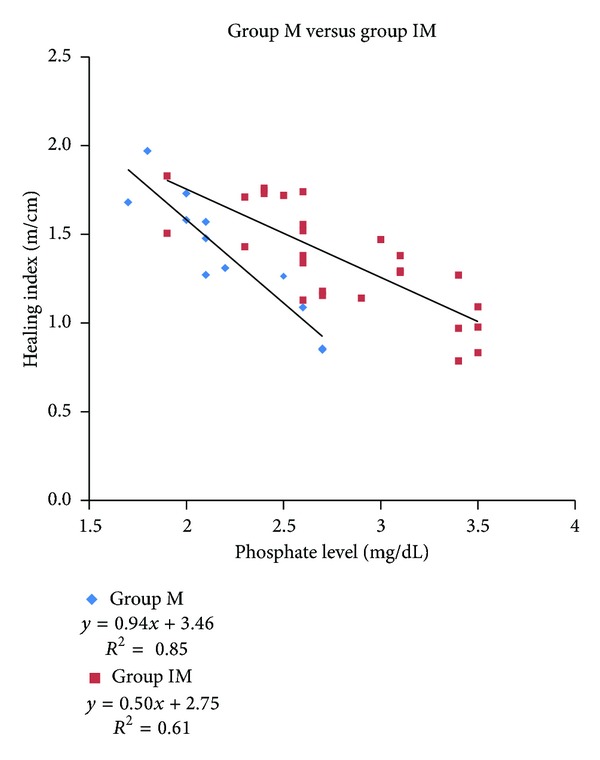
Healing index versus serumphosphate level in groups IM and M. (IM, immature; M,mature).

The correlation between HI and preoperative serum phosphate level of the patients was shown (Figure 2). There was a significant negative correlation between the two parameters (Spearman *γ* = −0.72, *P* = 0.01). Then the correlation of the parameters in groups IM and M was analyzed separately. The correlation equation and linear patterns in Figure 2 showed steeper negative slope in group M than in group IM (Figure 3).

The ROC curve analysis provided the optimal cut-off value for the serum phosphate level as being 2.6 mg/dL in group M and 2.3 mg/dL in group IM. In group M, the HI of those with a serum phosphate level higher than 2.6 mg/dL averaged 1.35 months/cm, while those with a serum phosphate level below 2.6 mg/dL averaged 1.74 months/cm. They were significantly different (*P* = 0.04). In group IM, the HI of those with a serum phosphate level higher than 2.3 mg/dL averaged 1.52 months/cm, while those with a serum phosphate level below 2.3 mg/dL averaged 1.82 months/cm. They were also significantly different (*P* = 0.03). Other biochemical parameters did not show any significant correlations with HI.

In group IM, there were 26 major complications and 22 minor complications in the 15 patients whose serum phosphate level was below 2.3 mg/dL and 15 major and 17 minor complications among the 12 patients whose serum phosphate level was above 2.3 mg/dL. In group M, there were 11 major and 9 minor complications among patients whose phosphate level was below 2.6 mg/dL and 3 major and 7 minor complications observed in patients who had a higher serum phosphate level than 2.6 mg/dL. Recurrent deformity, leg length discrepancy, refractures, and delayed union were the common bony complications (Table 4). The number of major bony complications was significantly different in two subgroups according to the phosphate cut-off level (*P* = 0.05 in group IM, *P* = 0.03 in group M; Mann-Whitney *U* test). However, there were no statistically significant differences in all other complications in all subgroups.

## 4. Discussion

Hypophosphatemic rickets is a disorder characterized by markedly low serum phosphate level and abnormal mineralization of bone. The defective bone mineralization results in rachitic changes at the growth plate and osteomalacia in trabecular and cortical bones [15]. The traditional treatment consists of controlling hypophosphatemia to prevent deformities of long bones and achieving normal growth with phosphate supplementation and pharmacologic doses of vitamin D or its derivatives. Despite adequate medical treatment, the growth response may be unsatisfactory and some patients remain unresponsive. In most cases, the deformities are not resolved and eventually require surgical interventions.

Previous authors reported various treatment modalities of deformity correction with or without limb lengthening in hypophosphatemic rickets patients [2–4, 10, 14] (Table 5). Song et al. [10] reported cases in which the patients had undergone acute or gradual corrections with external fixator with or without intramedullary nailing. And Matsubara et al. [3] demonstrated successful results of distraction osteogenesis with deformity corrections in their three skeletally mature hypophosphatemic rickets patients. However, Petje et al. [4] reported high recurrence rate of 90% after the first operation and 60% after the second procedure in their case series with long-term follow-up until skeletal maturity. Among them, Choi et al. [2] reported the significance of preoperative serum phosphate level as a key factor to obtain successful bone healing after deformity correction with concomitant limb lengthening procedures in a combined group of both 12 skeletally immature and 2 skeletally mature patients. They concluded that patients who cannot achieve and maintain the serum phosphate level at more than 2.5 mg/dL due to either severity of the disease or noncompliance with phosphate treatment are relatively contraindicated for concomitant lengthening because of delayed bone healing. In our study, we also observed negative correlation between the serum phosphate level and the healing index (Figure 3). The steeper slope observed in group M—compared to the slope of group IM—signified that the healing index of skeletally mature patients had a higher tendency to be affected by preoperative serum phosphate level. In other words, the lower level of serum phosphate brought forth higher likelihood of delayed union of the tibia. The difference in effectiveness of preoperative serum phosphate level on the two types of skeletal maturity was also proven by the cut-off values obtained via the ROC curve, which was evaluated to be 2.3 mg/dL in group IM and 2.6 mg/dL in group M. There have been previous studies that have discussed the effects of serum phosphate level on healing index or complications of corrective osteotomy in hypophosphatemic rickets patients. However, we believe that our study is the first report that investigated the relationship between serum phosphate level and healing index and postoperative complications in two comparative groups of skeletal maturity.

The study of Wigner et al. [1] reported that phosphate plays a critical role in normal skeletal development and responsiveness to bone morphogenetic proteins (BMP), which belong to the transforming growth factor-*β* superfamily. BMPs have been shown to play important roles in embryonic organ development, limb formation, and fracture healing including those that exhibit nonunion [1, 16–18]. In the context of fracture healing, BMPs bind to mesenchymal stem cells and promote their proliferation and differentiation into osteoprogenitor cells, making them truly osteoinductive [19]. Although it is clinically a known consensus that serum phosphate level affects bone consolidation and regeneration, only little is known about the physiological role of phosphate in skeletal development and repair. Bone formation and remodeling are energy consuming processes. The main sources of energy are in the form of adenosine phosphates and, to some extent, cyclic monophosphate, which is converted to adenosine triphosphate (ATP). Buchholz et al. [20] performed a study to investigate the correlation of ATP levels between bone metabolisms in the fractured tibiae of rabbit models using high-performance liquid chromatography. The mean ATP concentration in healthy cortical bone was significantly higher than that in the group with delayed healing and muscle-flap coverage. Thus, they concluded that the process of bone remodeling and repair was reflected by the level of ATP levels which are dependent on the level of serum phosphate. In this study, the clinical significance of the cut-off level of serum phosphate for safe healing could be assessed through the number of complications that occurred in the patients of each skeletal group with different values of serum phosphate. In group IM, the total number of major and minor complications observed in patients whose serum phosphate level is below the cut-off level of 2.3 mg/dL was 48 and that of major and minor complications seen in patients who maintained their serum phosphate above the cut-off level of 2.3 mg/dL was 32. In the same pattern, in group M, the total number of major and minor complications observed in patients whose serum phosphate level is below the cut-off level of 2.6 mg/dL was 20 and that of major and minor complications seen in patients who maintained their serum phosphate above the cut-off level of 2.6 mg/dL was 10. Also major bony complications in between subgroups of both group IM and group M were observed differently with statistical significances, indicating that serum phosphate level played a critical role for the bony healing process and affected bony complications significantly.

There were several limitations to this study. Firstly, this series involved a retrospective study of patients only whose tibia was osteotomized monofocally or bifocally. Many other patients who underwent surgeries for trifocal tibial corrective osteotomy or both tibial and femoral deformity corrections were excluded from the study. Thus, the cut-off serum phosphate level may not be representative of the entire population of hypophosphatemic rickets patients. However, gradual correction and lengthening were applied at the proximal osteotomy site and acute correction was applied at the distal osteotomy site in the bifocal deformity correction procedure. Healing index was assessed in reference to the result of gradual correction and lengthening applied at the proximal osteotomy site. So we believe the bias reflected on our result should be minimal. Secondly, other operative modalities such as intramedullary rod nailing and plate fixation were concomitantly used in a number of patients to prevent complications during or after deformity correction, hence causing bias in healing index.

In conclusion, this study demonstrated the ideal cut-off level of serum phosphate for safe healing after deformity correction and concomitant limb lengthening procedures in both skeletally mature and immature patients with hypophosphatemic rickets. And it should be carefully taken into consideration preoperatively according to the patient's skeletal maturity. If the patient's serum phosphate level does not reach the cut-off level but is willing to undergo deformity correction or leg lengthening, we suggest that the patient may try phosphate supplementation until his/her serum phosphate level is maintained above the cut-off level for prevention of any undesired complications and delayed union. We believe our findings may suggest more cautious guidelines for meticulous workup plans towards safe healing during preoperative planning and consultation with the patients and their guardians.

## Figures and Tables

**Table 1:** Radiologic data.

Group (*n*)	Deformity (*n*)	TFA (SD)			MAD (SD)		
		Pre-op	Post-op	Final	Pre-op	Post-op	Final
IM (27)	Genu varum (24)	19.9° (12.1)	4.5° (7.9)	5.6° (8.5)	3.5 cm (2.1)	1.0 cm (1.4)	1.7 cm (2.0)
	Genu valgum (3)	−10.0° (1.7)	−4.3° (6.1)	0.0° (3.5)	−1.6 cm (3.5)	−0.5 cm (1.3)	0.2 cm (0.7)

M (12)	Genu varum (10)	15.6° (13)	6.4° (5.8)	6.4° (6.0)	4.0 cm (3.2)	1.7 cm (2.1)	1.3 cm (2.0)
	Genu valgum (2)	−8.5° (4.9)	2.5° (7.8)	0.5° (7.8)	−2.9 cm (1.2)	0.2 cm (1.3)	−0.2 cm (0.3)

IM: immature; M: mature; TFA: tibiofemoral angle; MAD: mechanical axis deviation; final: measurement at the latest follow-up; SD: standard deviation; *n*: number of patients.

**Table 2:** Mean healing index and mean amount of lengthening.

Group (*n*)	Mean healing index (SD)	Mean lengthening (SD)
IM (27)	1.44 months/cm (0.53)	4.43 cm (2.61)
M (12)	1.68 months/cm (0.54)	4.96 cm (2.47)
*P* value	0.04	0.56

IM: immature; M: mature; *n*: number of patients; SD: standard deviation.

**Table 3:** Laboratory values.

Group (*n*)	Mean age (SD)	Mean phosphate (SD)	Mean ALP (SD)	Mean Ca (SD)
IM (27)	9.6 years (4.2)	2.78 mg/dL (0.47)	574.96 U/IL (197.28)	9.32 mg/dL (0.32)
M (12)	23.0 years (4.5)	2.21 mg/dL (0.34)	149.50 U/IL (65.41)	10.03 mg/dL (1.49)
*P* value	0.01	0.01	0.01	0.25

IM: immature; M: mature; ALP: alkaline phosphatase; *n*: number of patients; SD: standard deviation.

**Table 4:** Complications in two groups of patients.

Complications	Group IM (*n* = 27)		Group M (*n* = 12)	
	P < 2.3 mg/dL (*n* = 15)	P > 2.3 mg/dL (*n* = 12)	P < 2.6 mg/dL (*n* = 7)	P > 2.6 mg/dL (*n* = 5)
*Major *				
Bone				
Recurrent deformity	10	5	3	1
Refracture	1	1	1	—
Delayed union	1	—	2	—
Leg length discrepancy (>3 cm)	4	2	—	—
Total	**16**	**8**	**6**	**1**
*P* value	0.05		0.03	
Other				
Knee stiffness	2	1	1	—
Deep intramedullary infection	2	—	—	1
Equinus contracture	5	4	3	1
Peroneal nerve palsy	—	1	1	—
Patella dislocation	1	1	—	—
Total	**10**	**7**	**5**	**2**
*P* value	0.11		0.07	
*Minor *				
Bone				
Leg length discrepancy (<3 cm)	4	3	2	2
Angular deformity of less than 10°	5	3	1	1
Other				
Pin tract infection	11	9	5	3
Knee stiffness	2	2	1	1
Total	**22**	**17**	**9**	**7**
*P* value	0.44		0.32	

IM: immature; M: mature; *n*: number of patients; P: preoperative serum phosphate level; *n*: number of patients.

**Table 5:** Meta-analysis of deformity correction reports on hypophosphatemic rickets patients.

Author (year)	#	Age (range)	Segment and method of operation	Fixation method	Serum phosphate level (range)	Healing index	Complications
Rubinovitch et al. (1988) [14]	10	8 years (5–13)	26 tibiae, 16 femurs, 2 subtrochanteric shortening and compression plating	34 compression plates, 2 Sherman plates, and 2 staples	NR	NR	27% recurrence, anterior tibial compartment syndrome

Choi et al. (2002) [2]	14	13.9 years (3.2–22)	8 femurs, 4 tibiae in DC; 9 femurs, 19 tibiae in DCL	Ilizarov EF	2.3 mg/dL (1.3–3.0)	2.2 months/cm —DO	1 premature consolidation, 1 refracture, and 2 peroneal nerve palsies

Song et al. (2006) [10]	20	20 years (8–41)	20 femurs, 35 tibiae; 28 DCL, 27 acute DC	24 EF, 6 IM, 25 IM + EF	2.0 mg/dL (1.4–2.8)	2.6 months/cm—DO with EF 2.7 months/cm—DO with EF and IM	Recurrent deformity, equinus contracture, knee stiffness, and pin tract infection

Matsubara et al. (2008) [3]	3	33.2 years (24–46)	6 femurs, 4 tibiae; DCL	9 Ilizarov EF, 1 Heidelberg EF	2.1 mg/dL (2.10–2.3)	NR	None

Petje et al. (2008) [4]	10	8.3 years (4–11)	30 femurs, 35 tibiae; DC	53 EF, 19 Ilizarov, 18 Kirschner wires	2.1 mg/dL (2.0–2.7)	NR	Femoral fracture, pin tract infection, and 90% recurrence rate

Current study	12 M	23 years (18.3–31.2)	12 tibiae; 8 DC(L), 4 DCL	Ilizarov EF	2.2 mg/dL (1.7–2.8)	1.68 months/cm	Shown in Table 4
	27 IM	9.6 years (3.8–15.8)	27 tibiae; 12 DC(L), 15 DCL		2.6 mg/dL (1.9–3.5)	1.44 months/cm	

#: number of patients; M: group mature; IM: group immature; DO: distraction osteogenesis; DC: deformity correction; DC(L): acute correction and concomitant lengthening; DCL: gradual correction and lengthening; EF: external fixation; IM: intramedullary nailing; NR: not reported.
